# FSTL1 interacts with VIM and promotes colorectal cancer metastasis via activating the focal adhesion signalling pathway

**DOI:** 10.1038/s41419-018-0695-6

**Published:** 2018-05-29

**Authors:** Chuansha Gu, Xiaoyan Wang, Ting Long, Xia Wang, Yan Zhong, Yidan Ma, Zhiyan Hu, Zuguo Li

**Affiliations:** 1 Department of Pathology, School of Basic Medical Sciences, Southern Medical University, Guangzhou, China; 2 Guangdong Provincial Key Laboratory of Molecular Tumour Pathology, Guangzhou, China; 3 Department of Pathology, Nanfang Hospital, Southern Medical University, Guangzhou, China; 4 Department of Pathology, Shenzhen Hospital of Southern Medical University, Shenzhen, China

## Abstract

Follistatin-like protein 1 (FSTL1) has been reported to have both tumour-promoting and tumour-suppressive characters. However, the role of FSTL1 in colorectal cancer (CRC) remains unclear. Here we showed that FSTL1 expression was significantly up-regulated in CRC tissues compared with the paired normal tissues. In addition, the higher FSTL1 expression was associated with the infiltrating depth, lymph node metastasis and poor prognosis of CRC. Enhanced expression of FSTL1 distinctly increased cell migration and invasion in vitro, as well as promoting liver metastasis of CRC in vivo. Conversely, knockdown of FSTL1 expression significantly repressed invasion and metastasis of CRC. Mechanically, transcription factor Smad3 was involved in FSTL1 protein expression inducing by TGFβ1-Smad2/3 signalling. Furthermore, this effect of FSTL1 in promoting CRC progression was actualised via activating focal adhesions signalling pathway and regulating cytoskeleton rearrangement. We identified VIM, as an interactive protein of FSTL1, participated in FSTL1-mediated aggressive phenotype. We showed the role of FSTL1 in CRC and explored its transcription regulation and downstream signalling molecular mechanisms. In conclusion, our findings suggested that FSTL1 promoted CRC progression and metastasis, making it a novel target for diagnosis and prognostic evaluation of CRC.

## Introduction

Colorectal cancer (CRC) is one of the most common tumours and the third leading cause of cancer-related death in the world^1,2^. Although surgical techniques and adjuvant therapy have improved, the overall survival of patients with CRC has not improved obviously in recent years^3,4^. The main cause of mortality in patients with colorectal tumours is metastasis^5^, which is also a result of the accumulation of multiple genetic and epigenetic changes. Thus, a greater understanding of how molecular and cellular mechanisms of tumour progression combine to drive invasion and metastases in CRC is required.

Follistatin-like protein 1 (FSTL1), also refers as TSC-36 or FRP, is originally isolated in mouse osteoblastic MC3T3E1 cells as a cDNA that is up-regulated upon transforming growth factor-beta 1 (TGF-β1) stimulation^6^. It is a 308 amino acid secreted glycoprotein, which belongs to the osteonectin (SPARC) family of proteins containing both extracellular calcium-binding and follistatin-like domains. FSTL1 is a protein with multiple regulatory functions, including regulating organ tissue formation in embryos^7^, regenerating the heart’s cardiomyocytes^8^. In particular, FSTL1 plays an important role in the inflammatory response to various pathogens. It activates immune cells and promotes gene expression and releases of proinflammatory cytokines^9^, and promotes fibrosis after lung injury^10^.

To some extent, inflammation is a key inducement of tumour progression. In view of the special role of FSTL1 in inflammation, the effect of FSTL1 in tumour progression has attracted the interest of researchers. FSTL1 is up-regulated in a highly metastatic prostate cancer cell line and in most tumour tissue of glioblastoma multiforme patients^11,12^. FSTL1 promotes oncogenesis and metastasis in oesophageal squamous cell carcinoma by coordinating NF-kappa B and bone morphogenetic proteins (BMP) pathway^13^. Furthermore, it is a critical determinant governing cancer bone metastasis accompanied by expanding a population of pluripotent mesenchymal stem-like CD45^−^ALCAM^+^ cells derived from bone marrow^14^. On the contrary, this secreted glycoprotein is found to be down-regulated by using the primary cultures of metastatic and nonmetastatic clear-cell renal-cell carcinoma specimens for global gene-expression analysis^15^. FSTL1 is regarded as a tumour suppressor for inducing apoptosis of cancer cells in ovarian and endometrial cancers^16^. These results suggest that FSTL1 may have different regulatory effects in various tumour types. Therefore, the exact function of FSTL1 in the tumour needs to be further investigated.

So far, the role of FSTL1 in colorectal cancer progression and metastasis has not been well characterised. In the current study, we demonstrated the expression and clinical significance of FSTL1 in CRC samples, and then investigated the effects of aberrant expression on cellular biological behaviour by manipulating FSTL1 expression in CRC cells in vitro and in vivo. Importantly, we further explored the upstream and downstream regulation mechanism of FSTL1 in CRC progression and metastasis.

## Materials and methods

### Clinical samples and cell lines

Formalin-fixed paraffin embedded human colorectal carcinoma tissues (*n* = 130) for this study were obtained from the Department of Pathology, Nanfang Hospital, Southern Medical University, China. The clinical and pathological parameters of the patients were summarised in Table 1. The fresh surgically resected CRC tissues and matched adjacent normal tissues (*n* = 12) were immediately frozen in liquid nitrogen until the future study. In each case, a diagnosis of primary CRC had been made, and none of them had received any preoperative chemotherapy or radiotherapy. Blood samples (*n* = 15) of CRC patients were obtained from Nanfang Hospital, and normal samples of healthy donors (*n* = 13) were obtained from the Southern Medical University, China. All specimens were collected with the informed consent of patients. The study was approved by the ethics committee of Nanfang Hospital, Southern Medical University, China.

**Table 1:** The Relationship between FSTL1 expression and CRC clinicopathological features

Characteristics	Low	High	χ^2^ value	*P* value
Frequency	34	96		
Gender				
Male	20	59	0.073	0.787
Female	14	37		
Age				
≤58	12	47	1.891	0.169
>58	22	49		
Tumour size (diameter in cm)				
<5	23	66	0.039	0.843
≥5	11	29		
Differentiation				
Well	9	14	2.436	0.119
Moderate—Poor	25	82		
Depth of tumour invasion				
Mucosa-muscularis	14	18	6.805	0.009
Full-thickness	20	78		
Lymph node status				
Positive	7	40	4.833	0.028
Negative	27	56		
Dukes’ stage				
Dukes A+B	24	55	1.862	0.172
Dukes C+D	10	41		

Colorectal cancer cell lines, including Lovo, HT29, RKO, LS174T, Caco-2, DLD1, SW480, SW620, and a normal human foetal colonic mucosa cell line (FHC) were obtained from the Global Bioresource Centre (ATCC, USA). All cells were cultured in RPMI 1640 medium (Gibco, USA) supplemented with 10% foetal bovine serum (FBS) (Gibco, USA) at 37 °C in a humidified atmosphere with 5% CO_2_.

### Construction of stable cell lines

Lentivirus vector carrying the luciferase gene (Luc) and the human FSTL1 sequence (FSTL1) or the FSTL1-repressing shRNA sequence (GCCCAGTTGTTTGCTATCACT, FSTL1-sh) were purchased from Genechem (Shanghai, China). An empty vector (Vector) was used as control to FSTL1 overexpression. Lentivirus containing a scramble sequence (FSTL1-scramble) was used as control to FSTL1-shRNA. According to the manufacturer’s instructions, stable cell lines were established by transfection of CRC cells with these lentivirus vectors. Antibiotic-resistant transfected cells were selected via puromycin (Sigma, USA) administration in the culture medium. FSTL1 transfection efficiency was assessed by quantitative real-time PCR (qRT-PCR) and western blotting, respectively.

### RNA extraction and qRT-PCR

RNA extraction and qRT-PCR were performed, as previously described^17^. The primers for qRT-PCR ae described in Supplementary Table S1.

### Western blotting

Proteins extraction and western blotting were performed, as previously described^17^. The primary antibodies are displayed in Supplementary Table S2.

### Immunohistochemistry (IHC)

IHC was performed on paraffin sections of CRC tissues according to standard LSAB protocol (Dako), using primary antibodies against FSTL1, TGF-β1 (Proteintech, USA) respectively. The degree of staining in the sections was observed and scored independently by 2 pathologists. The percentage positivity of FSTL1 was scored from 0 to 3, with 0 for <10%, 1 for 10–30%, 2 for 31–50%, and 3 for >50%. The staining intensity was scored as 4-point scale: 0 (no staining), 1 (weak staining, light yellow), 2 (moderate staining, yellowish brown), and 3 (strong staining, brown). Subsequently, FSTL1 expression was calculated as the multiplication value of percentage positivity score and staining intensity score, which ranged from 0 to 9. The final expression level of FSTL1 was defined as low (0–4) and high (5–9)^18^.

### Enzyme linked immunosorbent assay (ELISA)

FSTL1 levels in the serum of CRC patients and cell supernatant were measured by ELISA using a commercially available kit (Huamei Biotech, China), as described by the manufacturer. The results were expressed in ng/ml, and the optical density of the samples was compared with the standard curves.

### Coimmunoprecipitation (CoIP)

Protein extracts from SW480 cells (500 μg/sample) were used to carry out the CoIP, as previously described^17^. The specific antibodies were displayed in Supplementary Table S2. Silver stain (Beyotime, China) was performed after immunoprecipitation WB and the different band was analysed by Quadrupole Mass Spectrometer (Thermo scientific, USA).

### Immunofluorescence and colocalization

Immunofluorescent staining was performed, as previously described^17^. The primary antibodies were displayed in Supplementary Table S2. 4, 6-Diamidino-2-phenylindole (DAPI) was used to stain the cell nucleus, and phalloidin (Cytoskeleton, USA) was used to stain the F-actin. The immunofluorescence and the colocalization of FSTL1 or FAK and VIM were recorded using the laser scanning confocal biological microscope. The images were analysed by using FV10-ASW 3.0 Viewer.

### Dual-Luciferase activity assay

Plasmid pGL3-WT containing wild-type FSTL1 promoter oligonucleotides (5′-AAGTCTGACTCCT-3′) and pGL3-MUT with mutant target site (5′-AGACTCAACTCCT-3′) was synthesised by Ruibiotech (Beijing, China). The sequences were predicted and designed according to bioinformatics analysis (http://jaspar.genereg.net/). 293 T cells and SW480 cells were seeded in 24-well plates (1 × 10^5^/well) and cultured 24 h before transfection. PGL3-basic, pGL3-WT, and pGL3-MUT plasmids were co-transfected with pRL-TK (Promega, USA) and pCMV3-smad3 respectively using Lipofectamine 3000 (Invitrogen, USA). pRL-TK (Promega) vectors were used as control. After 48 h, Luciferase activity was measured by the Dual-Luciferase Reporter Assay System (Promega, USA), as described by the manufacturer.

### Chromatin immunoprecipitation (ChIP)

ChIP assays were performed according to ChIP-IT Kit (Active Motif, USA). Antibody against smad3 or IgG (Cell Signaling Technology, USA) was used to precipitate the DNA-protein complex and subsequently elute the DNA from the antibody. Primers specific for the FSTL1 promoter were 5′-CTGCCATTCCAGCCTTTA-3′ (forward) and 5′-TGAGTGCCCACTGTTGTG-3′ (reverse).The immunoprecipitated DNA was examined by PCR.

### Cell migration and wound-healing assay, invasion and 3D culture assay, and cell proliferation assay

The migration and wound-healing assay, invasion and 3D culture assay, and cell proliferation of transfected CRC cells were determined as previously described^19^.

### Tumour metastasis assay in vivo

4–5 week-old Balb/C-nu/nu athymic mice (*n* = 5 per group) were obtained from Animal Centre of Guangdong Province (permit number: SYXK2016-0167). All animal experiments were approved by the Institutional Animal Care and Use Committee of Southern Medical University. Considering that FSTL1 may be affected by oestrogen, we chose male nude mice as the research objects. To investigate the tumour metastasis in vivo, 5 × 10^6^ of RKO cells stably expressed human FSTL1 sequence or empty vector carrying luciferase label in a volume of 75 μl in PBS were injected into spleen subcapsular, respectively. Multimodal Image Station System (Bruker, Germany) was used to obtain images of tumour progression and liver metastasis. The images were taken at 10 minutes after intraperitoneal injection of 150 μg/Kg D-Luciferin potassium salts (Synchem, USA). All the images were analysed by Bruker MI SE 721 Installer. After 8 weeks, the spleens and livers of nude mice were surgically removed after euthanasia, fixed in formalin (neutral buffered 10%), embedded in paraffin, and prepared into 3-μm sections for hematoxylin-eosin (HE) staining and IHC analysis.

### Statistical analysis

All statistical analyses were performed using the SPSS 19.0 (Abbott Laboratories, USA). All results were confirmed by statisticians in the Department of Health Statistics, Southern Medical University. The significance of correlation between the expression of FSTL1 and histopathological factors was determined using Pearson *χ*^2^ test. Survival analysis was carried out using the Kaplan–Meier method, and the log-rank test was used to compare the survival curves. Comparisons between groups were performed with a 2-tailed paired Student’s *t*-test. In vitro cell growth assay was tested using factorial design ANOVA. *P < 0.05, **P < 0.01 and ***P < 0.001 were considered statistically significant.

## Results

### FSTL1 expression in CRC correlates with tumour invasiveness and poor prognosis

**Fig. 1:**
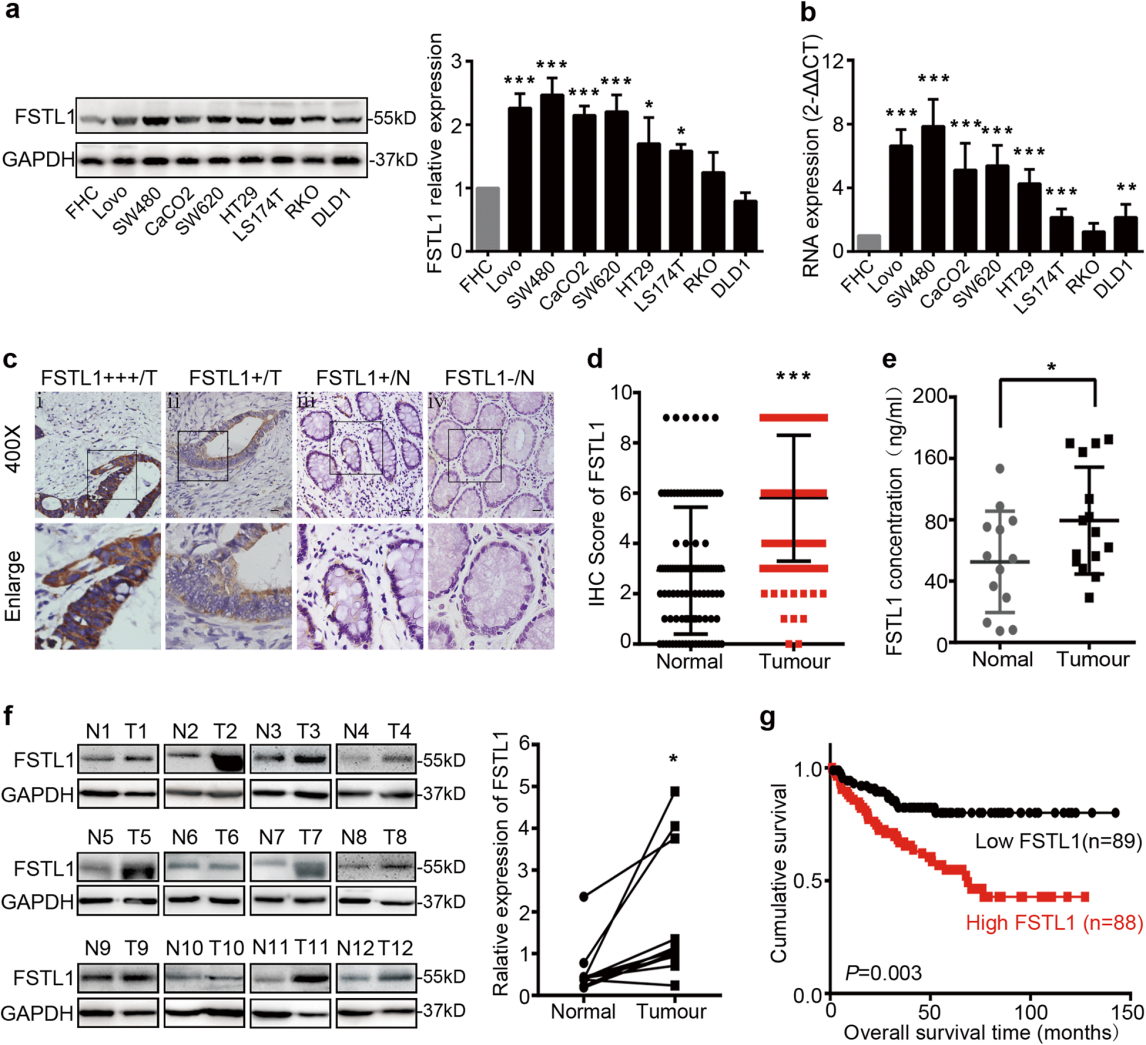
FSTL1 is up-regulated in CRC and correlates with poor prognosis. Expression analyses of FSTL1 protein and mRNA in CRC cell lines by **a** western blotting and **b** qPCR. FSTL1 protein levels were normalised to the relative expression of FHC. FSTL1 mRNA expression was quantified by qPCR and normalised to GAPDH. Error bars represent the mean ± S.D. (*n* = 3). **c** Immunohistochemistry (IHC) staining in 130 paraffin-embedded CRC tissues sections. (i) Strong expression (+++) in CRC. (ii) Weak expression (+) in CRC. (iii) Weak expression (+) in adjacent normal tissues. (iv) Scored negative for expression (−) in adjacent normal colorectal tissues. **d** Comparison of FSTL1 expression scores in CRC tissues (Tumour) with adjacent non-tumour tissues (Normal), *P* < 0.0001. **e** Elisa assay of FSTL1 in serum from healthy donors (Normal, *n* = 13) and CRC patients (Tumour, *n* = 15), *P* = 0.046. **f** Western blotting analysis of FSTL1 in 12 CRC tissues (T) and paired normal colorectal mucosa (*N*). Quantification of protein expression shown in right was normalised to GAPDH, *P* = 0.0178. **g** Kaplan–Meier survival analysis of GEO Database (GSE17536, *n* = 177) according to the expression of FSTL1, *P* = 0.003 (Log-rank test). *P < 0.05, **P < 0.01, ***P < 0.001

Western blotting and qRT-PCR were utilised to test the expression of FSTL1 in CRC cell lines and the normal colonic mucosa cell line (FHC). Our results revealed that FSTL1 was up-regulated in all the 8 CRC cell lines at the protein and mRNA level. (Fig. 1a, b). Immunohistochemistry (IHC) staining was performed in 130 paraffin-embedded CRC tissues sections. As shown in Fig. 1c, different levels of FSTL1 expression were observed in these tissue samples. According to classification as detailed described in method, overexpression of FSTL1 was detected in 96 out of 130 (73.8%) CRC cases compared with 34 out of 103 (33.0%) in adjacent non-tumour tissues. FSTL1 expression score was also higher in tumour tissues (*P* < 0.0001, Fig. 1d). The correlation between FSTL1 expression level and CRC clinical features was further analysed (Table 1). The data indicated that overexpression of FSTL1 was closely related to depth of tumour invasion (*P* = 0.009), and lymph node metastasis (*P* = 0.028). Moreover, FSTL1 expression in 12 CRC tissues (T) and paired normal colorectal mucosa (N) was detected by western blotting. As a secreted protein, FSTL1 level in the serum was measured by ELISA. Consistent with the tissues samples, higher FSTL1 expression of serum was found in 15 CRC patients compared with 13 healthy donors (*P* = 0.046, Fig. 1e). As shown in Fig. 1f, an increase in FSTL1 expression was observed in CRC tissues compared with adjacent normal tissues (*P* = 0.0178).

In addition, to investigate whether the different levels of FSTL1 expression in CRC are related to patient’s prognosis, we performed bioinformatic analysis of NCBI GEO Database (GSE17536, *n* = 177). Kaplan–Meier survival analysis revealed that patients with a higher level of FSTL1 expression had a worse clinical outcome (*P* = 0.003, Fig. 1g). These observations demonstrate that FSTL1 is up-regulated in CRC and correlates with its depth of tumour invasion, lymph node metastasis and poor prognosis.

### FSTL1 promotes CRC cells migration and invasion in vitro

**Fig. 2:**
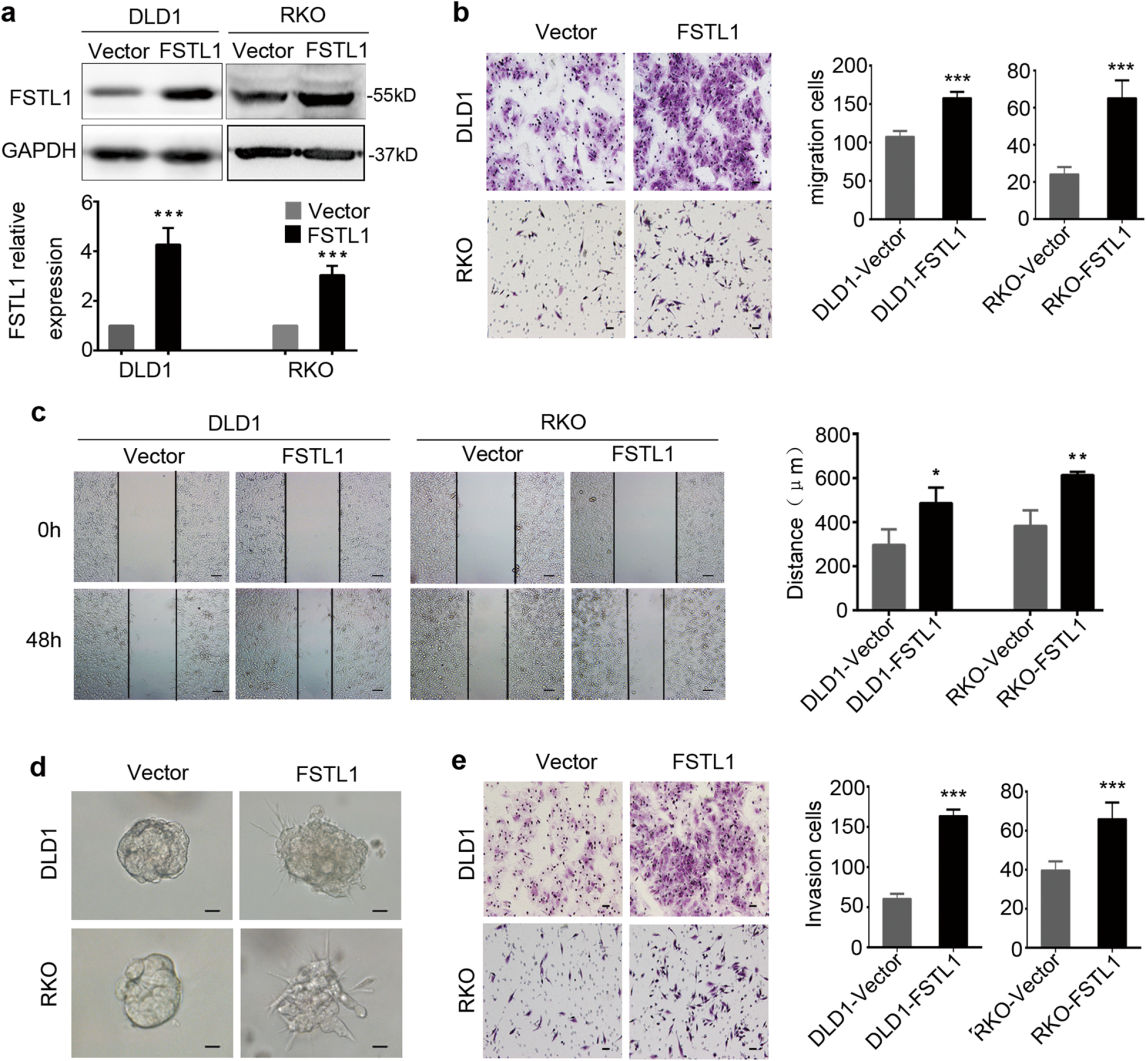
FSTL1 promotes CRC cells migration and invasion in vitro. **a** DLD1 and RKO cells were stably transfected with vector (Vector) or FSTL1 lentivirus (FSTL1). Western blotting analysis was performed to detect the expression of FSTL1 (upper panel). FSTL1 protein levels were normalised to the relative expression of Vector group respectively, *P* < 0.001 (lower panel). Error bars represent the mean ± S.D. (*n* = 3). **b** The migratory cells of trans-well assay were counted under microscope in five randomly selected fields (scale = 50 μm). Representative photographs (left) and quantification (right) are shown, both *P* < 0.0001. Error bars represent the mean ± S.D. (*n* = 5). **c** Wound healing assay was performed to evaluate the motility in DLD1 cells, *P* = 0.03 and RKO cells, *P* = 0.0052 (scale = 100 μm). Error bars represent the mean ± S.D. (*n* = 3). **d** Three-dimensional culture assays was performed to observe the effect of FSTL1 on cell invasion (scale = 20 μm). FSTL1 overexpressing cells were extended protuberances to the Matrigel matrix, and the control cells formed tight spherical colonies. **e** The invasive cells of Matrigel-coated Boyden chamber invasion assay were counted under microscope in five randomly selected fields (scale = 50 μm). Representative photographs (left) and quantification (right) are shown, both *P* < 0.0001. Error bars represent the mean ± S.D. (*n* = 5). *P < 0.05, **P < 0.01, ***P < 0.001

**Fig. 3:**
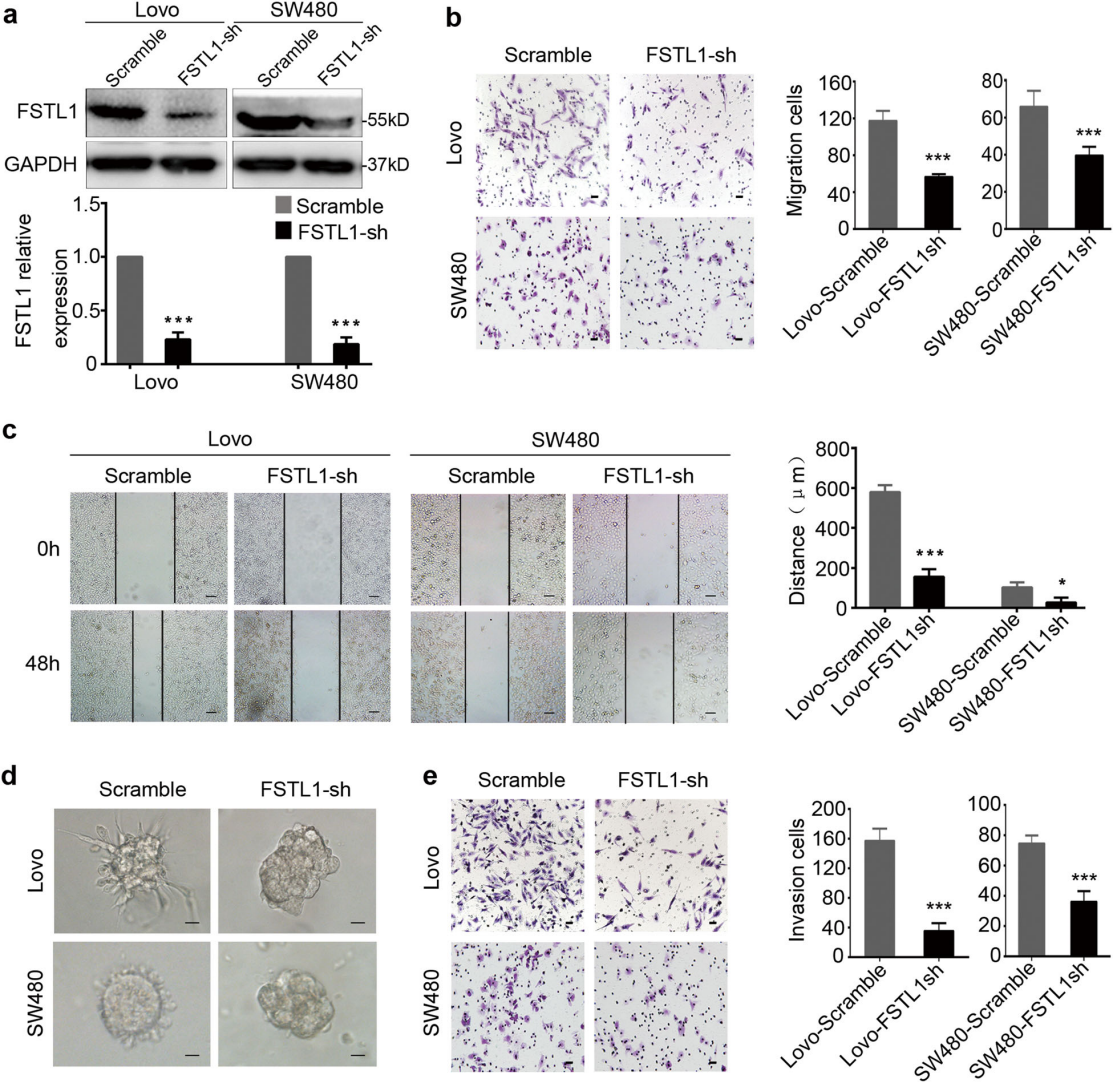
FSTL1 konckdown attenuates CRC cells migration and invasion in vitro. **a** Lovo and S480 cells were stably transfected with scramble sequence (Scramble) or FSTL1-repressing shRNA sequence lentivirus (FSTL1-sh). Western blotting analysis was performed to detect the expression of FSTL1 (upper panel). FSTL1 protein levels were normalised to the relative expression of Scramble group respective, *P* < 0.001 (lower panel). Error bars represent the mean ± S.D. (*n* = 3). **b** The migratory cells of trans-well assay were counted under microscope in five randomly selected fields (scale = 50 μm). Representative photographs (left) and quantification (right) are shown, both *P* < 0.0001. Error bars represent the mean ± S.D. (*n* = 5). **c** Wound healing assay was performed to evaluate the motility in Lovo cells, *P* = 0.0001 and SW480 cells, *P* = 0.0203 (scale = 100 μm). Error bars represent the mean ± S.D. (*n* = 3). **d** Three-dimensional culture assays was performed to observe the effect of FSTL1 on cell invasion (scale = 20 μm). Knockdown of FSTL1 inhibited the invasive phenotype. **e** The invasive cells of Matrigel-coated Boyden chamber invasion assay were counted under microscope in five randomly selected fields (scale = 50 μm). Representative photographs (left) and quantification (right) are shown, both *P* < 0.0001. Error bars represent the mean ± S.D. (*n* = 5). *P < 0.05, **P < 0.01, ***P < 0.001

To gain insight into the effect of FSTL1 on cellular behaviour in CRC tumourigenesis and progression, we generated DLD1-FSTL1 and RKO-FSTL1 cell lines that were stably overexpressed full-length FSTL1. Furthermore, Lovo-FSTL1-sh and SW480 FSTL1-sh cell lines that stably expressed FSTL1-shRNA were made. The expression level of FSTL1 in stable lines was verified by western blotting analysis and qRT-PCR (Figs. 2a and 3a, Supplementary Figure S1a and S2a).

Since FSTL1 expression was related to CRC invasion depth and lymph node metastasis as described, trans-well and wound-healing assays were performed. As shown in Fig. 2b, c, the migration and motility ability of DLD1-FSTL1 cells (*P* < 0.0001, *P* = 0.03) and RKO-FSTL1 cells (*P* < 0.0001, *P* = 0.0052) were enhanced compared with the control cells. Matrigel-coated Boyden chamber invasion assay revealed that overexpression of FSTL1 significantly promoted the invasive ability of CRC cells in vitro (both *P* < 0.0001, Fig. 2e). Conversely, knockdown of FSTL1 slowed the migration and motility ability of Lovo-FSTL1-sh cells (*P* < 0.0001, *P* = 0.0001) and SW480 FSTL1-sh cells (*P* < 0.0001, *P* = 0.0203), and decreased the invaded cell numbers (both *P* < 0.0001, Fig. 3b, c, e). Moreover, to further validate the effect of FSTL1 on cell invasion, we performed three-dimensional culture assays. Different stable cells were seeded in three-dimensional Matrigel cultures at a same density and their colony shapes were examined 10 days later. Expectedly, FSTL1 over-expressing cells were highly invasive and extended protuberances to the Matrigel matrix, and the control cells formed tight spherical colonies (Fig. 2d). On the contrary, knockdown of FSTL1 inhibited the invasive phenotype (Fig. 3d). Neither overexpression nor knockdown of FSTL1 showed any significant difference in the capacity of CRC cells proliferation by CCK-8 assay (Supplementary Figure. S1b and S2b). These data indicate that FSTL1 promotes CRC cellular migration and invasion in vitro, but has no effect on CRC cells proliferation.

### FSTL1 overexpression enhances CRC metastasis in vivo

**Fig. 4:**
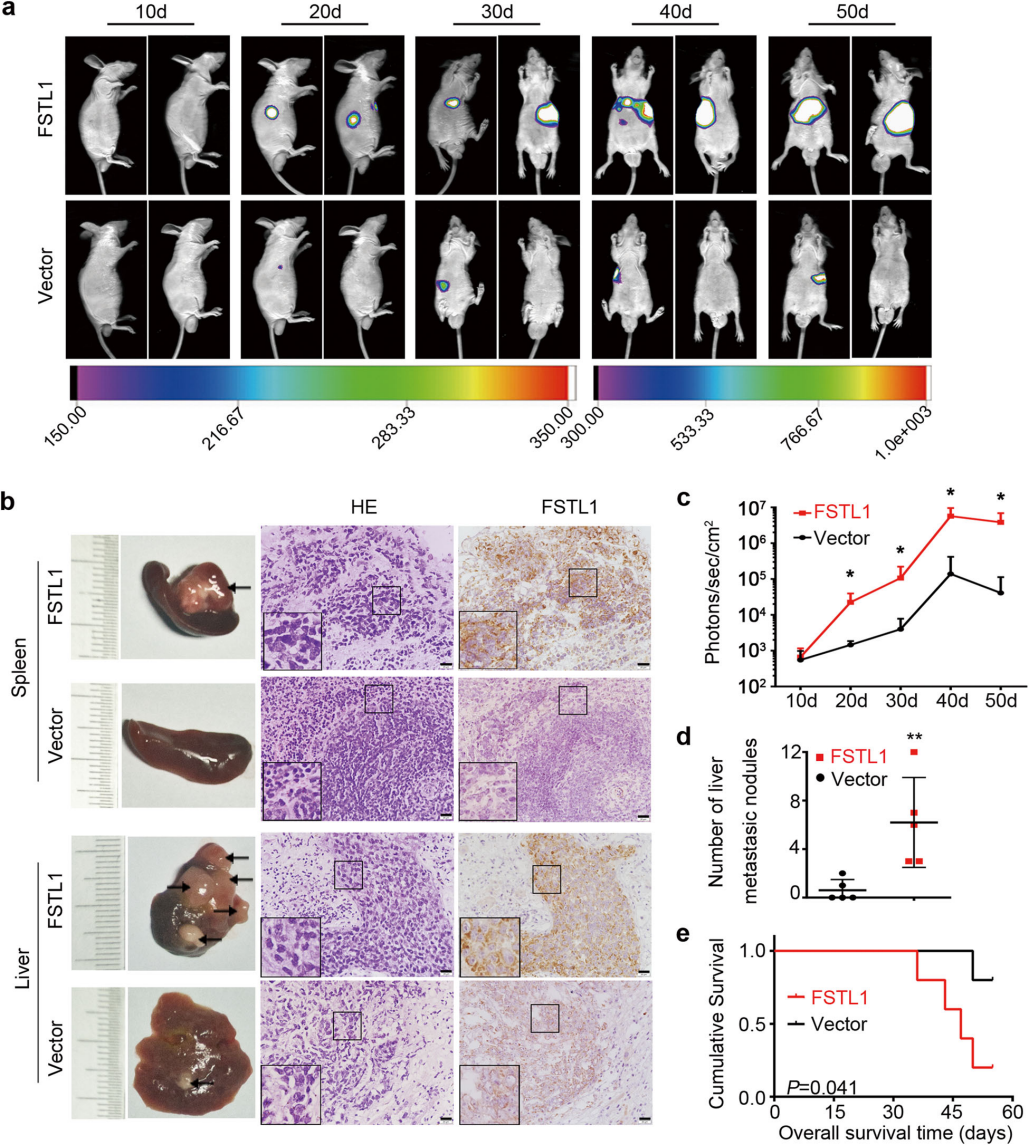
FSTL1 promotes CRC cells metastasis in vivo. In total 5 × 10^6^ of RKO-FSTL1 or RKO-Vector stable cells were injected into spleen subcapsular respectively of nude mice to evaluate tumour metastasis. Multimodal Image Station System (Bruker, Germany) was used to obtained images of tumour progression and liver metastasis. **a** Two representative images of each group are shown at every 10 days after injection (*n* = 5). **b** Spleens and livers derived from indicated cells were harvested at 8 weeks after spleen subcapsular injection. The typical pictures of xenograft tumours of spleen and metastatic nodules of liver in gross morphology and HE or FSTL1 staining are shown (×400, scale = 20 μm). The image in black pane of the lower left corner is a partial enlarged detail. **c** The photons/s/cm^2^ of individual mice were recorded and analysed at every 10 days after injection, each *P* < 0.05. Error bars represent the mean ± S.D. (*n* = 5). **d** The number of liver metastatic nodules in individual mice was counted under the microscope and analysed, *P* = 0.010. (*n* = 5)**. e** Log-rank test was performed to estimate the relevance of FSTL1 expression and survival time of nude mice, *P* = 0.041. (*n* = 5). *P < 0.05, **P < 0.01, ***P < 0.001

The effect of FSTL1 on tumour metastasis was assessed by spleen subcapsular injection of human CRC cells into nude mice. Fig. 4a showed two typical pictures of each group on every 10 days after injection. In order to observe more easily, the left lateral position of the mouse was used at 10 and 20 days, and the prone position was used after 30 days. Xenograft tumours in spleen and metastatic nodules in liver were earlier detected in the group of FSTL1 overexpression (FSTL1) compared with those in the control group (Vector). 8 weeks after injection, larger tumours of spleens and more liver metastatic nodules were observed in the FSTL1 group in gross morphology (Supplementary Figure. S3). As shown in Fig. 4b, IHC staining confirmed that the tumours derived from FSTL1 group exhibited higher FSTL1 expression levels than tumours derived from control cells. Significant difference was revealed in photons/s/cm^2^ between the FSTL1 group and Vector group from 20 days after injection (Fig. 4c). Moreover, the number of liver metastatic foci of the FSTL1 group was more than the Vector group (*P* = 0.010, Fig. 4d). In addition, overexpression of FSTL1 shortened the survival time of nude mice (*P* = 0.041, Fig. 4e). These results reveal that overexpression of FSTL1 significantly enhances metastasis of CRC cells in vivo.

### TGFβ1-Smad2/3 signalling pathway regulates the expression of FSTL1 through activating the transcriptional activity of Smad3 in human CRC

**Fig. 5:**
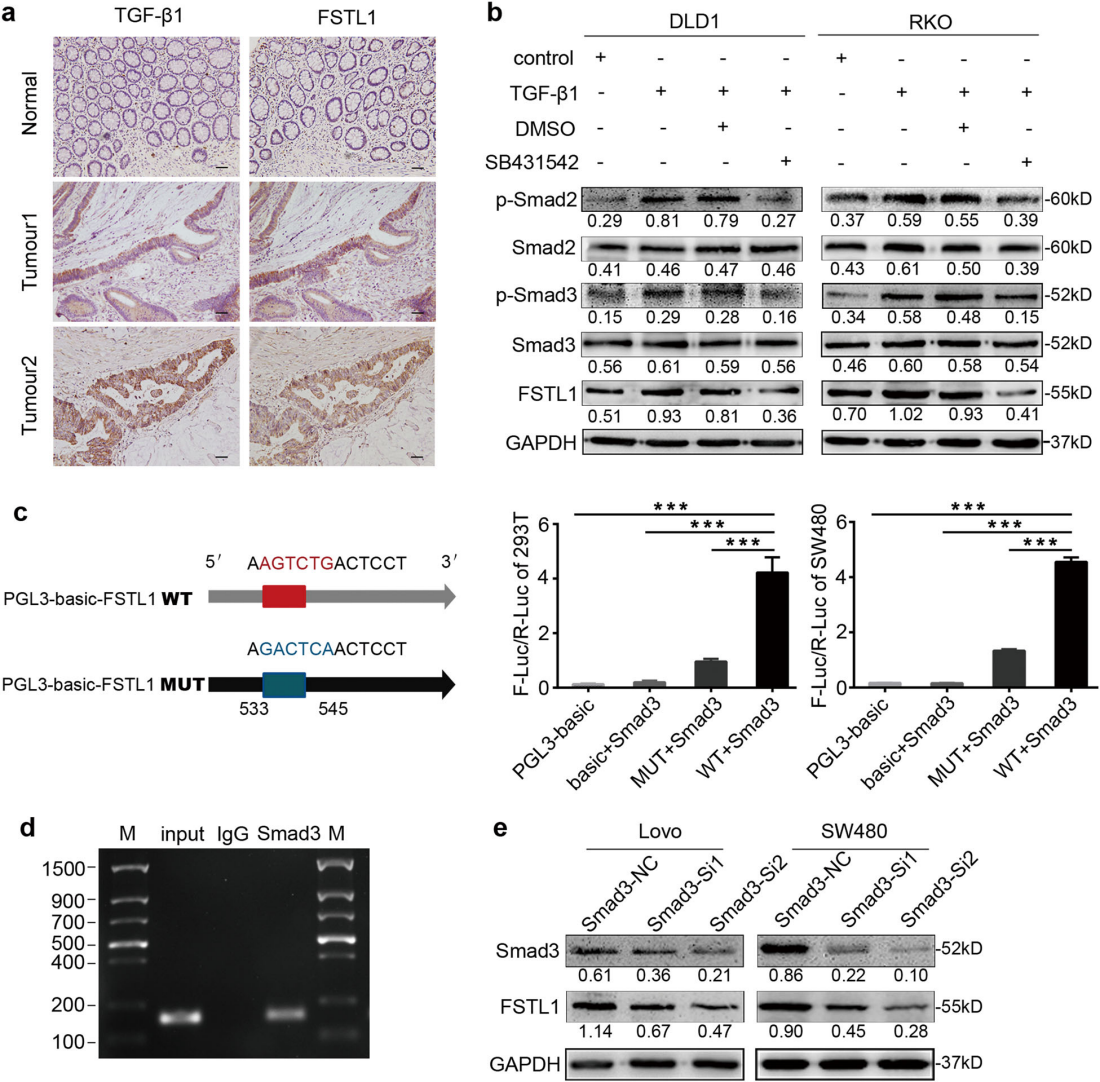
TGFβ1-Smad2/3 signalling pathway regulates the expression of FSTL1 through activating the transcriptional activity of Smad3 in human CRC. **a** Representative TGF-β1 and FSTL1 immunohistochemistry staining photographs of normal tissue (Normal) and tumour tissue samples (Tumour 1, Tumour 2), (×200, scale = 50 μm). The TGF-β1 and FSTL1 were spatially correlated. **b** TGF-β1 activated Smad2/3 signalling and enhanced FSTL1 expression in DLD1 and RKO cells by western blotting. SB431542 attenuated the expression of P-smad2, P-smad3 and FSTL1. **c** Schematic of the FSTL1 promoter luciferase construct is depicted with the locations of binding site and the sequences of mutation (left). Luciferase activities in HEK293T and SW480 cells were examined after transfecting with Smad3 (right), each *P* < 0.001. Error bars represent the mean ± S.D. of the ratio of firefly and renilla luciferase signals (*n* = 3). **d** Smad3 binding on the promoter region of FSTL1 was assessed by ChIP assay. Immunoprecipitation from SW480 cells using Smad3 antibody or rabbit immunoglobulin G (IgG). PCR from the IP samples using FSTL1-specific primers. **e** Western blotting analysis of Smad3 and FSTL1 in Lovo and SW480 cells, after using smad3-siRNA mediated RNA interference. *P < 0.05, **P < 0.01, ***P < 0.001

Although FSTL1 was initially identified as TGF-β1-induced protein in a murine cell line, the molecular mechanisms underlying its expression in human CRC is not yet understood. As shown in Fig. 5a, FSTL1 and TGF-β1 protein expression levels in consecutive paraffin-embedded slice of human CRC tissue were detected by IHC. The bioinformatic analyses indicated that there was positive correlation between FSTL1 and TGF-β1. (http://r2.amc.nl, Supplementary Figure. S4a). CRC cell lines DLD1 and RKO with lower FSTL1 expression were treated with recombinant human TGF-β1 (rhTGFβ-1). Western blotting analysis indicated that TGF-β1 enhanced Smad2/3 phosphorylation (P-Smad2/3) and FSTL1 expression. While blocking TGF-β signal transduction by SB431542, a specific inhibitor of Smad2/3, TGF-β1-induced FSTL1 protein expression decreased (Fig. 5b). Meanwhile, neither overexpression nor knockdown of FSTL1 significantly changed the phosphorylation levels of smad2 and smad3 in stable CRC cells (Supplementary Figure S4b).

To further explore the upstream mechanism of FSTL1 expression, potential transcription factors promoting were analysed by bioinformatic algorithms. It showed that Smads (smad2/smad3/smad4) is the predicted transcription factor with binding site on the FSTL1 promoter at 533 to 545 bp (including GTCT box) in the JARSPAR databases. Smad3 can be combined directly with DNA, whereas Smad2 can not, because Smad2 has 2 amino acid fragments more than Smad3^20^. So we performed dual-luciferase activity assay to verify whether smad3 participated in regulating the expression of FSTL1. As shown in Fig. 5c, compared with other groups, the ratio of firefly and renilla luciferase signals increased significantly in WT group which was transfected with plasmid pGL3-WT containing wild-type FSTL1 promoter oligonucleotides (*P* < 0.0001). In addition, ChIP assays demonstrated that smad3 bound to FSTL1 promoter (Fig. 5d). Subsequently, Western blotting was carried out in Lovo and SW480 cells using Smad3-siRNA mediated RNA interference. The expression of FSTL1 reduced after knockdown of Smad3 (Fig. 5e). Collectively, these data reveal that TGFβ1-Smad2/3 signalling pathway regulates FSTL1 expression through activating the transcriptional activity of transcription factor Smad3.

### FSTL1 activates the focal adhesion signalling pathway and regulates cytoskeleton rearrangement

**Fig. 6:**
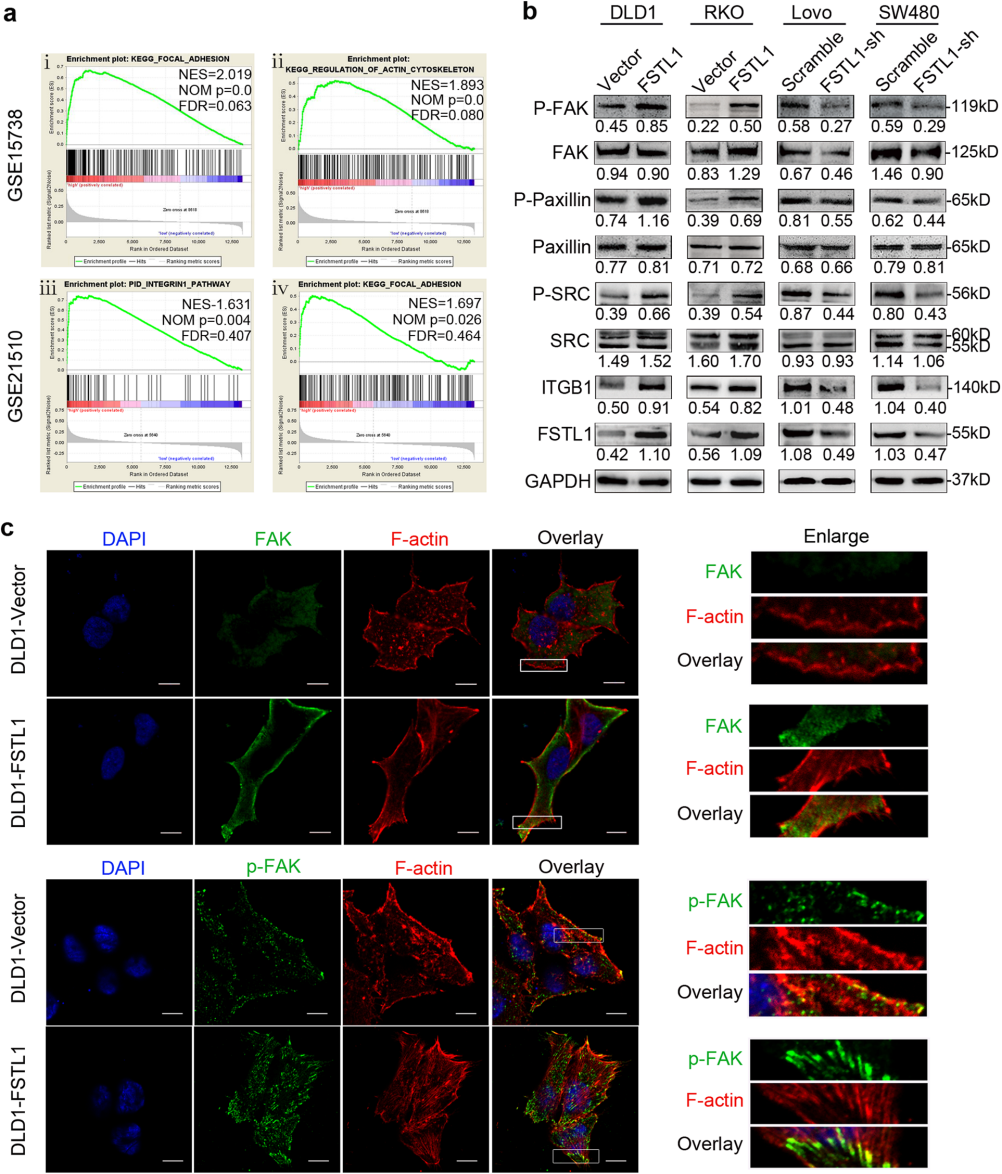
FSTL1 activates the focal adhesion signalling pathway and regulates cytoskeleton rearrangement. **a** Gene Set Enrichment Analysis (GSEA) was performed to exploring FSTL1 effect in CRC. (i) KEGG-Focal adhesion, normalised enrichment score (NES) = 2.019, nominal *P* value (NOM *P*) = 0.000, false discovery rate (FDR) = 0.063. (ii) KEGG-Regulation of actin cytoskeleton, NES = 1.893, NOM *P* = 0.000, FDR = 0.080. (iii) PID-Integrin1 pathway, NES = 1.631, NOM *P* = 0.004, FDR = 0.407. (iv) KEGG-Focal adhesion, NES = 1.697, NOM P = 0.026, FDR = 0.464. **b** Western blotting analysis was performed to detect the expression of FSTL1 and the main members of focal adhesion signalling pathway in indicated stable cells. Phosphorylated FAK at Tyr-397, Phosphorylated Paxillin at Tyr-118, and Phosphorylated SRC at Tyr-418. **c** Immunofluorescent staining was performed to observe the expression of FAK (green, upper panel), p-FAK (green, lower panel) and actin cytoskeleton in DLD1-Vector and DLD1-FSTL1 cells (×1800, scale = 10 μm). Phalloidin (red) stains F-actin, DAPI (blue) stains nuclei. Right column are the partial enlarged details from the white pane of corresponding original images

To understanding the molecular mechanism of FSTL1 in CRC progression, the CRC data were downloaded from GEO public database and Gene Set Enrichment Analysis (GSEA) was carried out. KEGG-Focal adhesion, KEGG-Regulation of actin cytoskeleton and PID-Integrin1 pathway gene sets were positive enriched (Fig. 6a). Western blotting analysis was performed to detect the levels of FSTL1 and the main members of focal adhesion signalling pathway, including phospho-FAK (Tyr-397), FAK, phospho-Paxillin (Tyr-118), Paxillin, phospho-SRC (Tyr-418), SRC and Integrin-β1. As shown in Fig. 6b, accompanied by changes of FSTL1 protein expression, these major molecules have occurred corresponding changes. Next, we explored whether FSTL1 had an effect on regulation of actin cytoskeleton. As shown in Fig. 6c,d and Supplementary Figure. S5, immunofluorescent staining showed that the pattern of F-actin changed from spike-like squiggles or short nailed protuberances to long stress fibres in the FSTL1 overexpression cells compared with control cells. Furthermore, the expression of FAK enhanced in FSTL1 overexpression group compared with the control group. Interestingly, phospho-FAK concentrated from the cytoplasm to the edge and formed more focal adhesions in the FSTL1 overexpression cells. These results suggest that FSTL1 takes part in cell adhesion and regulate cytoskeleton rearrangement via activating focal adhesion signalling pathway.

### VIM is an interactive factor of FSTL1

**Fig. 7:**
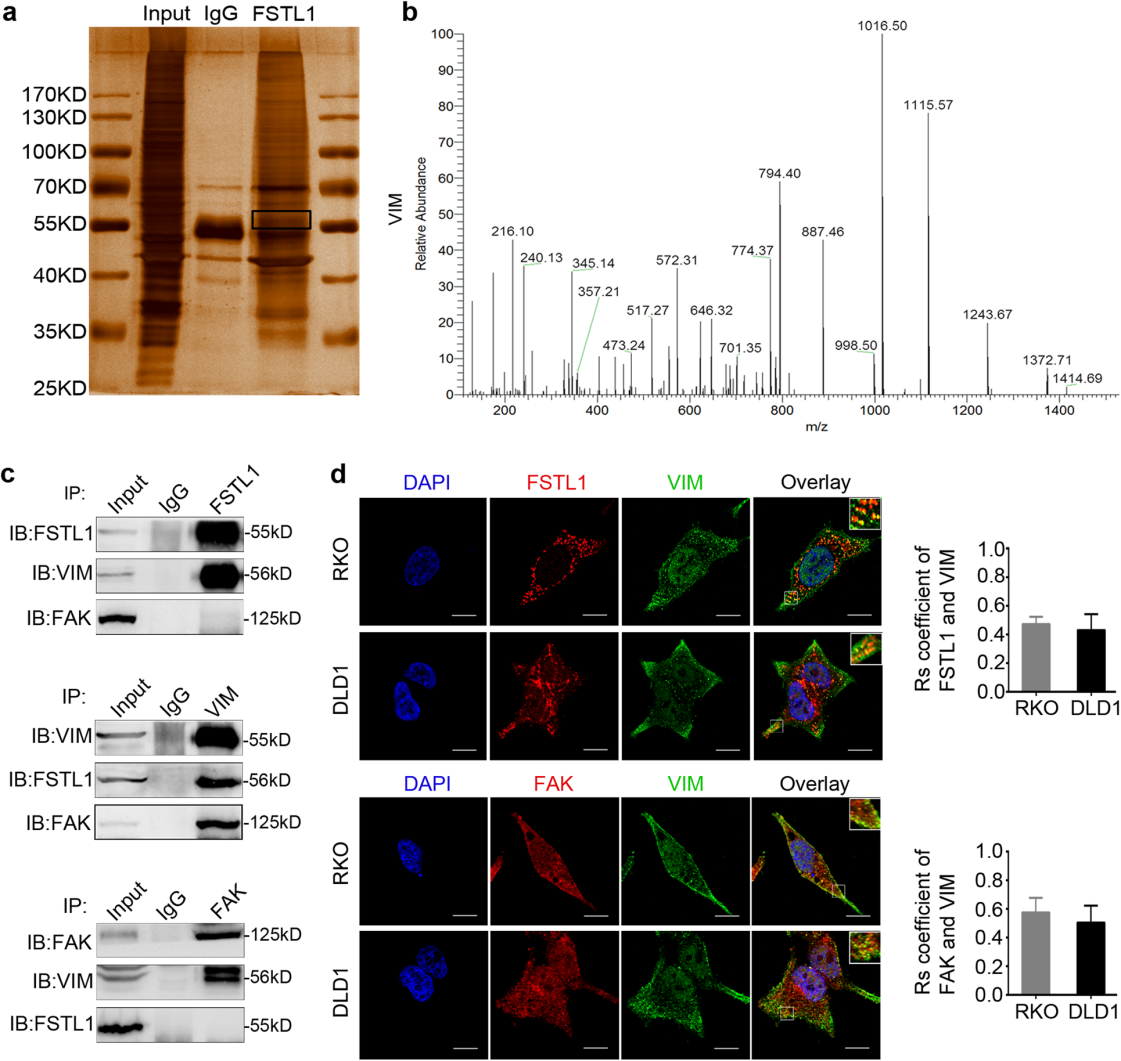
VIM is an interactive factor of FSTL1. **a** Immunoprecipitation and silver staining was performed by using the whole proteins SW480 cells with anti-FSTL1 antibody. **b** The spectrogram of differential protein band is identified as VIM (Quadrupole Mass Spectrometer). **c** Coimmunoprecipitations were performed to validate the interaction among FSTL1, VIM, and FAK in SW480 cells. **d** FSTL1 (red, upper panel) or FAK (red, lower panel) co-localizate with VIM (green) in RKO and DLD1 cells was assessed by laser-scanning confocal microscopy, respectively (×1800, scale = 10 μm). DAPI (blue) stains nuclei. The image in white pane of the upper right corner of overlay is a partial enlarged detail. Error bars in the histogram represent the mean ± S.D. of the colocalisation coefficients of VIM and FSTL1 (upper panel) or the colocalisation coefficients of VIM and FAK (lower panel) in RKO cells and DLD1 cells (*n* = 10)

To identify unknown interactive molecules of FSTL1, the whole proteins of whole-cell lysate from SW480 cells was extracted and immunoprecipitated with FSTL1 antibody (Fig. 7a). As shown in Fig. 7b, the most clearly differential protein band was identified as VIM by liquid chromatography-mass spectrometry. According to the results, we conjectured that FSTL1, VIM and FAK might form a complex. Thus protein samples were confirmed by CoIP with antibodies against FSTL1 or VIM (Fig. 7c). The IF assays validated localisation of VIM and its partial colocalisation with FSTL1 (Fig. 7d, upper panel). The colocalisation coefficients of VIM and FSTL1 in RKO cells and DLD1 cells are 0.473 ± 0.023 (*n* = 10) and 0.431 ± 0.050 (*n* = 10), respectively. Previous studies have reported that VIM enters into focal adhesions sites and regulates cell adhesion^21,22^. The interaction between VIM and FAK was proved by CoIP and colocalisation (Fig. 7d, lower panel). The colocalisation coefficients of VIM and FAK in RKO cells and DLD1 cells are 0.576 ± 0.045 (*n* = 10) and 0.505 ± 0.023(*n* = 10), respectively.

## Discussion

In the light of the high morbidity and mortality of colorectal cancer, efforts to elucidate new biomarkers and more effective therapies are still imperative in order to improve survival for CRC patients. FSTL1 has been reported in the fields of inflammation, postischemia vascular remodelling, immune regulation and autoimmune diseases in recent years^9^. Oncomine analysis (https://www.oncomine.org) showed that the expression of FSTL1 varies according to the organs and systems. It is up-regulated in some tumour types, including brain and CNS cancer, colorectal cancer, pancreatic cancer, prostate cancer and so on. So far, the explicit expression and particular function of FSTL1 in human CRC still remain obscure. In the present study, we investigated the expression of FSTL1 in a clinical cohort with 130 cases. Our data verified that the overexpression of FSTL1 in CRC tissues was associated with the depth of tumour infiltration and the distant metastasis. A significant correlation was found between FSTL1 overexpression and CRC patient poor survival. Furthermore, due to FSTL1 is a secreted protein, it is detectable in the blood serum of patients. Our data showed that FSTL1 in serum of CRC patients was higher than that in health donors. These observations support the notion that FSTL1 may serve as a valuable biomarker in tumorigenesis and progression of CRC.

As an inflammatory cytokines, FSTL1 could be induced in vitro in osteoblasts, adipocytes, chondrocytes, and human fibroblast-like synoviocytes by IL-1β, TNFα, and IL-6. The NF-ƘB pathway was involved in the induction of FSTL1 gene transcription^23,24^. Moreover, using an inducible Akt1 transgenic mouse model, researchers found that Fstl1 protein and transcript expression are increased by Akt activation in the heart^25^. However, the regulation of FSTL1 expression in human tumours had hitherto not fully elucidated. As already mentioned, FSTL1 was cloned as a TGF-β1 inducible gene. Meanwhile, TGF-β1 is well known to be regarded as a metastasis inducer, participating in malignant progression and angiogenesis^26^. Therefore, we verified the consistency of the expressions of FSTL1 and TGF-β1 in CRC tissues. More importantly, we firstly demonstrated in some detail that TGFβ1-Smad2/3 signalling pathway regulated FSTL1 expression through activating the transcriptive activity of transcription factor Smad3, which combined with the promoter of FSTL1 directly in human CRC cells. These observations might present an opportunity to exploit such a mechanism for intervention of FSTL1 and therapeutic gain.

FSTL1 has proven to be able to enhance CRC cells migration and invasion in vitro and promote liver metastasis of CRC in vivo in the present study. We further investigated its downstream mechanism and interactive molecular targets. KEGG-Focal adhesion, KEGG-Regulation of actin cytoskeleton and PID-Integrin1 pathway gene sets were positive enriched by GSEA. Focal adhesion kinase (FAK) is a cytoplasmic protein tyrosine kinase that is overexpressed and activated in several invasive tumours^27^. FAK is at the intersection of various signalling pathways promoting cancer cell motility^28^, invasion^29^, cell survival^30^, and epithelial-mesenchymal transition (EMT)^31^. The Tyr-397 phosphorylation and kinase activity of FAK are substantiated to be essential for the invasive phenotype^32^. Our results demonstrated that FSTL1 activated FAK focal adhesion signalling pathway in CRC cells, and followed by regulating cytoskeleton rearrangement. It suggested that focal adhesion signalling was involved in the FSTL1-mediated acceleration of CRC progression.

We further explored the proteins interacting with FSTL1 via a protein–protein interaction technique. Among the candidate proteins, VIM was identified as a novel target of FSTL1. VIM, a major constituent of the intermediate filament family of proteins, is widely known as a marker for EMT. It correlates with accelerated tumour growth, invasion, and poor prognosis. In addition, it is considered to be a regulator of focal adhesions (FAs) and motility^33^. VIM assembles into focal adhesions and it is required for FAK activity and localisation at the cellular leading edge of motile cancer cells^22,34,35^. VIM assembly into focal adhesions is an important factor of cell adhesion strength and its overexpression enhances FAs turnover^36^. The results of CoIP showed that there was no interaction between FSTL1 and FAK, while VIM had interaction with FSTL1 and FAK, respectively. Therefore, we speculated that VIM might act as a media. The detailed mechanisms about how the interaction of FSTL1 and VIM is involved in focal adhesion signalling pathway in CRC cells will be interesting subjects for the future study.

In summary, we demonstrated that FSTL1 was up-regulated and had a close relationship with poor outcome in CRC. The overexpression of FSTL1, inducing by TGFβ1-Smad2/3 signalling pathway, functionally promoted CRC cells migration, invasion, and metastasis by combining with VIM and activating focal adhesion signalling pathway consequently. Therefore, our data provide evidence for FSTL1 being regarded as a detectable biological marker that reflects malignant degree and evaluates survival in CRC.

## Electronic supplementary material

SI summary

Supplementary Information
