# Supplementary material for: FSTL1 interacts with VIM and promotes colorectal cancer metastasis via activating the focal adhesion signalling pathway
Source: Cell Death Dis. 2018 May 29;9(6):654. doi: 10.1038/s41419-018-0695-6 (PMC5974179; doi:10.1038/s41419-018-0695-6)
Supplement: Supplementary file 1 — SI summary [file 41419_2018_695_MOESM1_ESM.docx]

**Figure. S1** Overexpression of FSTL1 has no effect on CRC cells proliferation.

**Figure. S2** Knockdown of FSTL1 has no effect on CRC cells proliferation.

**Figure. S3** FSTL1 promotes CRC cells metastasis *in vivo*.

**Figure. S4** The protein expression of FSTL1 and TGF-β1 is positively correlated.

**Figure. S5** FSTL1 activates the focal adhesion signalling pathway and regulates cytoskeleton rearrangement.

**Table S1** Primer sequences for qRT-PCR (5' to 3')

**Table S2** Antibodies used for Western blotting, Coimmunoprecipitation and Immunofluorescence
