# Supplementary material for: FSTL1 interacts with VIM and promotes colorectal cancer metastasis via activating the focal adhesion signalling pathway
Source: Cell Death Dis. 2018 May 29;9(6):654. doi: 10.1038/s41419-018-0695-6 (PMC5974179; doi:10.1038/s41419-018-0695-6)
Supplement: Supplementary file 2 — Supplementary Information [file 41419_2018_695_MOESM2_ESM.docx]

**
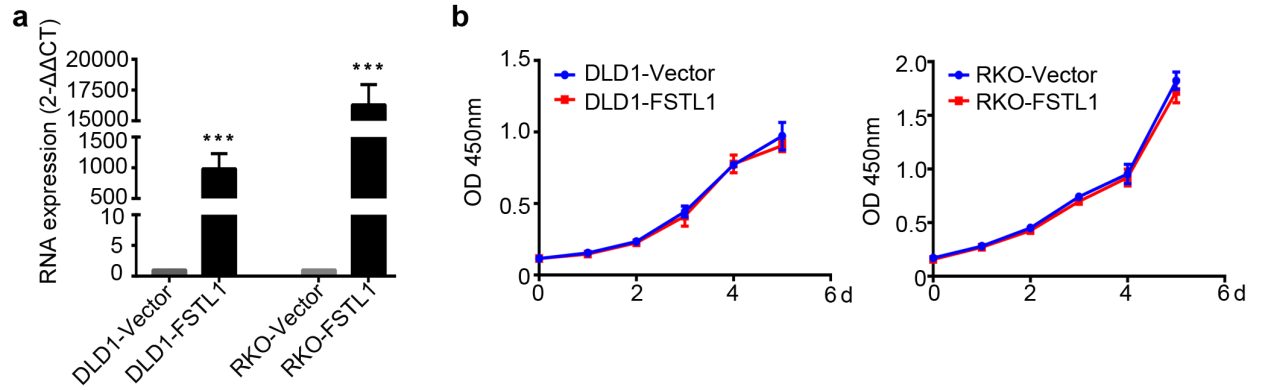
Figure. S1** Overexpression of FSTL1 has no effect on CRC cells proliferation. **a** Expression analyses of FSTL1 mRNA in DLD1 and RKO stable cells by qRT-PCR. FSTL1 mRNA expression was normalized to GAPDH, both *P*<0.0001. Error bars represent the mean ±S.D. (n=3). **b** CCK8 assay of FSTL1 overexpression group compared with control group at each corresponding time point. Error bars represent the mean ±S.D. (n=4). **P*<0.05, ***P*<0.01, ****P*<0.001.


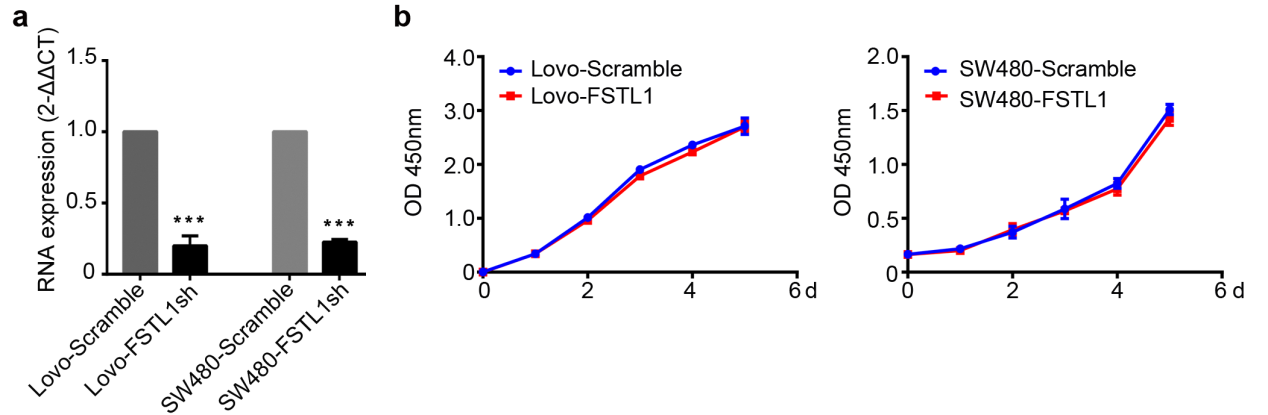


**Figure. S2** Knockdown of FSTL1 has no effect on CRC cells proliferation. **a** Expression analyses of FSTL1 mRNA in Lovo and SW480 stable cells by qRT-PCR. FSTL1 mRNA expression was normalized to GAPDH, both *P*<0.0001. Error bars represent the mean ±S.D. (n=3). **b** CCK8 assay of FSTL1 knockdown group compared with control group at each corresponding time point. Error bars represent the mean ±S.D. (n=4). **P*<0.05, ***P*<0.01, ****P*<0.001.


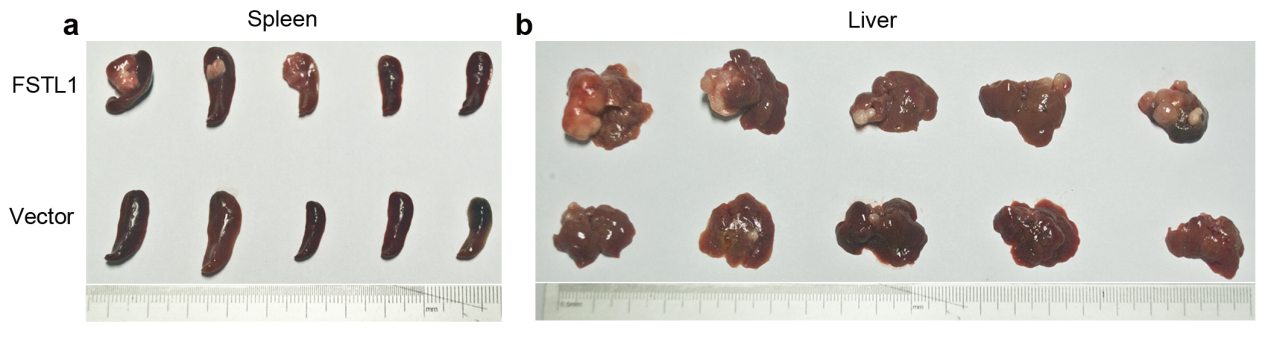


**Figure. S3** FSTL1 promotes CRC cells metastasis *in vivo*. Gross morphology of spleens (left) and livers (right) derived from RKO-FSTL1 and RKO-Vector cells at 8 weeks after spleen subcapsular injection.

**
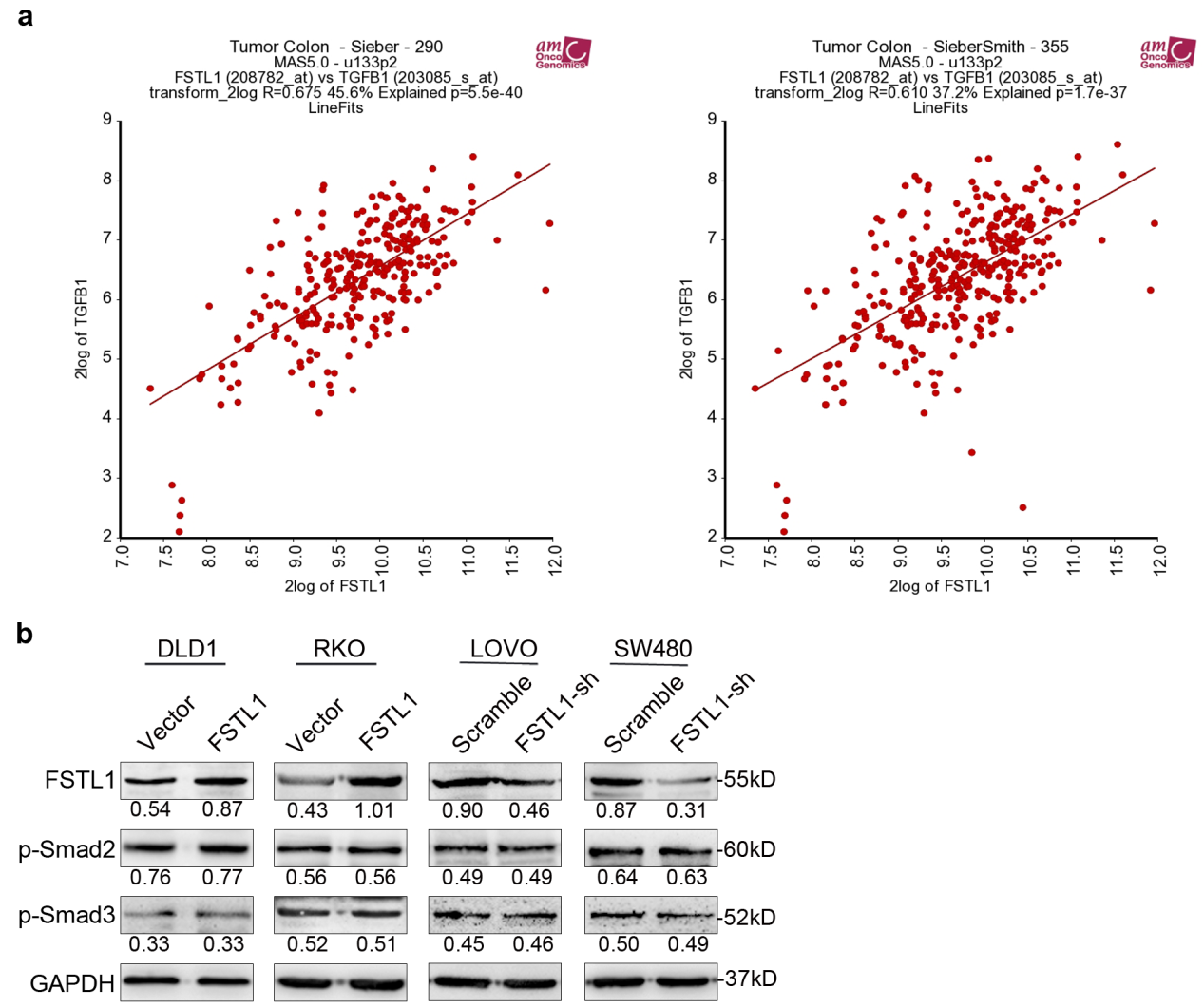
**

**Figure. S4** The protein expression of FSTL1 and TGF-β1 is positively correlated. **a** Correlation analysis of the protein expression of FSTL1 and TGF-β1 was carried out by bioinformatics prediction (<http://r2.amc.nl>). **b** Western blotting analysis of P-Smad2 and P-Smad3 in indicated stable CRC cells had been constructed.

**
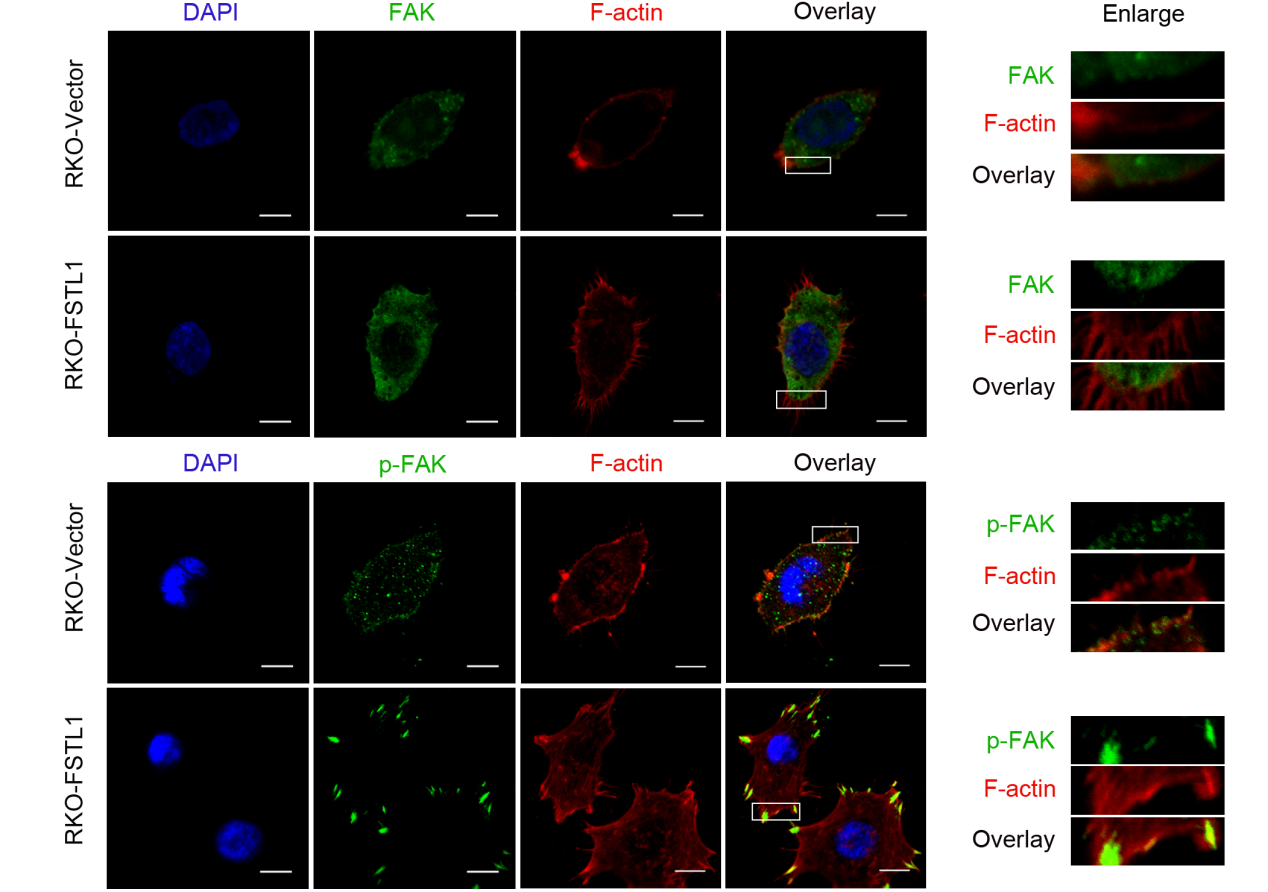
Figure. S5** FSTL1 activates the focal adhesion signalling pathway and regulates cytoskeleton rearrangement. Immunofluorescent staining for FAK (green, upper panel), p-FAK (green, lower panel) in RKO cells (1800×, scale=10μm). Phalloidin (red) stains F-actin, DAPI (blue) stains nuclei. Right column are the partial enlarged details from the white pane of corresponding original images.

**Table S1** Primer sequences for qRT-PCR (5' to 3')

| **Gene** | **Forward primer** | | **Reverse primer** |
| --- | --- | --- | --- |
| FSTL1 | TCTGTGCCAATGTGTTTTGTGG | TGAGGTAGGTCTTGCCATTACTG | |
| GAPDH | GGAGCGAGATCCCTCCAA AAT | GGCTGTTGTCATACTTCTCATGG | |

**Table S2** Antibodies used for Western blotting, Coimmunoprecipitation and Immunofluorescence

| **Antibody** | **Manufacturer** | **Country** | **dilution** |
| --- | --- | --- | --- |
| **Western blotting** | | |  |
| FSTL1 | Proteintech | USA | 1:500 |
| P-Smad2 | Cell Signalling Technology | USA | 1:500 |
| P-Smad3 | Cell Signalling Technology | USA | 1:500 |
| Smad2 | Cell Signalling Technology | USA | 1:1000 |
| Smad3 | Cell Signalling Technology | USA | 1:1000 |
| TGF-β1 | Proteintech | USA | 1:1000 |
| p-FAK | Abcam | UK | 1:1000 |
| FAK | Cell Signalling Technology | USA | 1:1000 |
| p-Paxillin | Bioworld | USA | 1:500 |
| Paxillin | Abcam | UK | 1:500 |
| p-SRC | Abcam | UK | 1:1000 |
| SRC | Proteintech | USA | 1:500 |
| ITGB1 | Abcam | UK | 1:500 |
| GAPDH | Proteintech | USA | 1:2000 |
| **Coimmunoprecipitation** | | |  |
| FSTL1 | Proteintech | USA | 1:500 |
| VIM | Proteintech | USA | 1:2000 |
| FAK | Cell Signaling Technology | USA | 1:1000 |
| **Immunofluorescence** | | |  |
| FSTL1 | Proteintech | USA | 1:100 |
| VIM | Proteintech | USA | 1:1000 |
| P-FAK | Abcam | UK | 1:200 |
| FAK | Abcam | UK | 1:200 |
